# Beyond Intercalation Chemistry for Rechargeable Mg Batteries: A Short Review and Perspective

**DOI:** 10.3389/fchem.2018.00656

**Published:** 2019-01-15

**Authors:** Zhirong Zhao-Karger, Maximilian Fichtner

**Affiliations:** ^1^Helmholtz Institute Ulm, Ulm, Germany; ^2^Institute of Nanotechnology, Karlsruhe Institute of Technology, Karlsruhe, Germany

**Keywords:** magnesium battery, magnesium battery electrolyte, conversion-type cathode, magnesium-sulfur batteries, organic cathode

## Abstract

Rechargeable magnesium (Mg) batteries are an attractive candidate for next-generation battery technology because of their potential to offer high energy density, low cost, and safe use. Despite recent substantial progress achieved in the development of efficient electrolytes, identifying high-performance cathode materials remains a bottleneck for the realization of practical Mg batteries. Due to the strong interaction between the doubly charged Mg^2+^ ions and the host matrix, most of the conventional intercalation cathodes suffer from low capacity, high voltage hysteresis, and low energy density in Mg based battery systems. Alternatively, the thermodynamically favorable conversion reaction may circumvent the sluggish Mg^2+^ diffusion kinetics. In this review, the focus will be laid on promising cathodes beyond the typical intercalation-type materials. We will give an overview of the recent emerging Mg systems with conversion-type and organic cathodes.

## Introduction

Efficient and cost-effective electrical energy storage system is regarded as the feasible solution for the implement of the renewable energy and carbon-free transportation. Lithium ion batteries (LIBs) have emerged as the state-of-the-art technology for powering today's portable electronics and have also been introduced for e-mobility and stationary storage applications. However, with the constantly increasing demands for long-lasting customer electronics and extended driving range for electric vehicles (EVs), the current advanced LIBs are not able to meet the energy requirements and the safety issue remains unresolved (Etacheri et al., 2011; Yoo et al., 2014; Manthiram, 2016). In addition, the globally rapid growing market of EVs and grid-scale electricity storage give rise to concerns about the availability of the raw materials such as cobalt and lithium, which are essential components in current commercial LIBs. Besides the limited natural abundances, the production of both lithium and cobalt is geographically restricted as most of lithium reserves are located in South America while around 90% of the world's reserves of cobalt are concentrated in Congo and Zambia, which appear to be politically unstable (Vaalma et al., 2018). With these critical concerns, increasing attention has been paid to sustainable electrode materials and post-lithium battery technologies (Armand and Tarascon, 2008; Larcher and Tarascon, 2015). In particular, owing to the low cost and natural abundance of sodium resources, sodium-ion batteries (SIBs) have been extensively investigated, and regarded as the most competitive alternative power technology to the current LIBs (Vaalma et al., 2018). Battery systems based on multivalent metals such as magnesium, zinc, calcium, and aluminum have been recently proposed as next-generation technologies (Xu et al., 2012; Muldoon et al., 2014; Lin et al., 2015; Ponrouch et al., 2016). Multivalent metals undergo beyond one-electron redox reactions and could in principle provide higher volumetric energy densities than the monovalent Li-ion and Na-ion batteries. The resource availability of these multivalent ions renders such battery systems potentially economic for large-scale applications (Muldoon et al., 2014).

Mg battery has emerged as one of the most promising candidates because of the ideal features of Mg to be used as a metal anode. Mg has the low reduction potential (−2.37 V vs. SHE), high theoretical volumetric capacity (3833 mAh cm^−3^), high abundance in the earth crust and dendrite-free metal deposition (Gregory, 1990; Aurbach et al., 2001; Jäckle and Groß, 2014). The key challenges of realization of Mg batteries lie in the development of efficient electrolytes with high electrochemical stability and identifying suitable cathode materials offering high voltage and high capacity (Yoo et al., 2013; Mohtadi and Mizuno, 2014; Saha et al., 2014; Shterenberg et al., 2014; Bucur et al., 2015; Erickson et al., 2015; Song et al., 2016; Canepa et al., 2017). In the past decades, significant progress has been made in the development of the electrolyte which exhibits favorable electrochemical properties, is chemically compatible with both Mg anode and cathode materials and non-corrosive to the common cell components. However, the searching for high-performance cathode material remains the most critical hurdle toward a practical Mg full cell.

In the light of the success in commercialization of LIBs, intercalation cathode materials have been of great interest to be employed for Mg batteries due to their potentially high cell voltage, high energy density, and cycling stability. However, most of the host cathodes used for LIBs has been proven to exhibit poor capability of Mg-ion storage although Mg ion has a similar ionic radii as Li ion (i.e. 86 and 90 pm, respectively). In fact, the major factors hindering Mg^2+^ ion intercalation stem from the high charge density of the divalent Mg ion, which is more than doubled than that of the monovalent Li^+^ (120 vs. 54 C mm^−3^). The strong electrostatic forces between the Mg ions and the surrounding anions in crystal matrix induce the intrinsically slow kinetics of Mg^2+^ ion insertion and diffusion within the host cathodes. Consequently, when Mg^2+^ ions intercalate, the conventional oxide based cathode materials suffer from low capacity, high voltage hysteresis and undergo irreversible conversion reactions at the cathode surface. Use of “soft” chalcogenides would improve the performance, but it compromises the high voltage of the intercalation cathode yielding a low energy density for the full cell (Aurbach et al., 2000).

Conversion-type electrodes have been proposed as alternative candidates for Mg batteries because of the high theoretical capacities with multi-electron transfer and low cost (Zhao-Karger and Fichtner, 2017; Zhang et al., 2018). The thermodynamically favorable conversion reaction may circumvent the sluggish Mg^2+^ insertion/diffusion kinetics and can be a potential alternative to the intercalation chemistry. Conversion electrodes undergo a redox reaction that involves a chemical transformation by breaking and creating chemical bonds. Analogously to the concepts in Li-ion chemistry, the conversion reactions for Mg batteries can be generally expressed as

(1) $$\begin{array}{r} \text{Type A }\left(\text{exchange reaction}\right):\text{ M}{\text{X}}_{\text{a}}+\text{a}/2\text{M}{\text{g}}^{2+}+\text{a}{\text{e}}^{-}⇄\text{M}+\text{ a}/2\text{Mg}{\text{X}}_{2} \end{array}$$

(2) $$\begin{array}{r} \text{Type B }\left(\text{combination reaction}\right):\text{ M}{\text{g}}^{2+}+{\text{X}}_{\text{a}}+2{\text{e}}^{-}⇄\text{Mg}{\text{X}}_{\text{a}} \end{array}$$

For the Type A reaction, MX_*a*_ can typically be transition metal halide, oxide, chalcogenide, nitrides or phosphides. As well-acknowledged in the research in LIBs, Type A electrode materials in general suffer from poor electronic conductivity, large voltage hysteresis, large volume change and low conversion efficiency (Cabana et al., 2010; Nitta et al., 2015; Wu and Yushin, 2017; Yu et al., 2018). As the reaction 1 involves the formation of the intermediate insertion phase and the fully converted new phases, high ion mobility within the solid electrodes is a prerequisite for enabling the efficient conversion. Due to the fundamental diffusion limitation of Mg-ion in solid-state, it is challenging to explore a proper Type A cathode for Mg batteries. Attempts with sulfides such as CoS and CuS have been taken, where a low reversible capacity was exhibited (He et al., 2015; Duffort et al., 2016; Xiong et al., 2018). At an elevated temperature e.g., 150°C, CuS could deliver a high initial discharge capacity of 550 mAh g^−1^, but it decreased to 200 mAh g^−1^ from the second cycle (Duffort et al., 2016).

By comparison, conversion reaction 2 basically indicates a relatively less complicated reaction process by combining two simple reactants to form a single-product. The Type B cathodes include chalcogens (e.g., S, Se, and Te) and halogens (e.g., Br_2_ and I_2_). Among them, S and Se have been considered as particularly promising cathode materials for various battery systems including Li and multivalent metal-based systems (Cohn et al., 2015; Gao et al., 2016; Zhao-Karger and Fichtner, 2017; Yang et al., 2018). Oxygen is conceptually also a Type B cathode, but the technological issues to be addressed are fundamentally different from those with the common solid cathodes. Mg-air batteries therefore are not included in this review.

In addition, organic compounds are attractive electrode materials owing to their promising electrochemical performance and distinct advantages in structural diversity, modification flexibility, sustainability and environmental friendliness (Schon et al., 2016; Bhosale et al., 2018). The low volumetric energy density of organic cathodes in LIBs would be greatly compensated by pairing with a Mg anode. In this review, we will focus on promising cathodes for Mg batteries beyond the typical intercalation-type materials which have been reviewed elsewhere (Jantsch and Zemek, 1952; Yoo et al., 2013; Mohtadi and Mizuno, 2014; Muldoon et al., 2014; Saha et al., 2014; Bucur et al., 2015; Huie et al., 2015; Song et al., 2016; Canepa et al., 2017; Erickson et al., 2017). In particular, we will discuss the current development and future prospects of emerging Mg systems with conversion type and organic cathodes which were enabled and improved by the advanced Mg electrolytes.

## Conversion-type Cathodes for Full Mg Batteries

### Mg-S and Mg-Se Batteries

Metal-sulfur (S) battery chemistries are receiving increasing attentions because of the high theoretical capacity (1,675 mAh g^−1^), low toxicity and abundance of sulfur as a cathode candidate. Lithium-sulfur (Li-S) battery, possessing a high theoretical energy density of 2,800 Wh l^−1^ and, has been suggested as low-cost post Li-ion system. However, use of the Li-metal anode induces severe safety issues associated with growth of Li dendrite during the cycling of the batteries. In contrast, dendrite-free deposition of Mg enables Mg metal an ideal anode material. Coupling a sulfur cathode with a Mg metal anode, based on a two-electron conversion reaction of Mg^2+^ + S + 2e^−^ ⇄ MgS, Mg-S battery offers a theoretical cell voltage of 1.77 V and an energy density of 1,722 Wh kg^−1^ and 3,200 Wh l^−1^, respectively, which renders Mg-S battery an attractive option for future battery generations (Aurbach et al., 2001). The key challenge in the realization of Mg-S batteries has been to develop a sulfur compatible electrolyte with favorable electrochemical properties. In fact, great progress has been achieved in formulating effective Mg electrolytes, which has also facilitated the research on Mg-S batteries. Electrolyte generally plays a vital role in battery chemistry and performance. In the following sections, we will give an overview of recent studies on Mg-S batteries carried out with different type of electrolyte solutions.

#### With Bis-Amide Based Electrolyte

**Figure 1:**
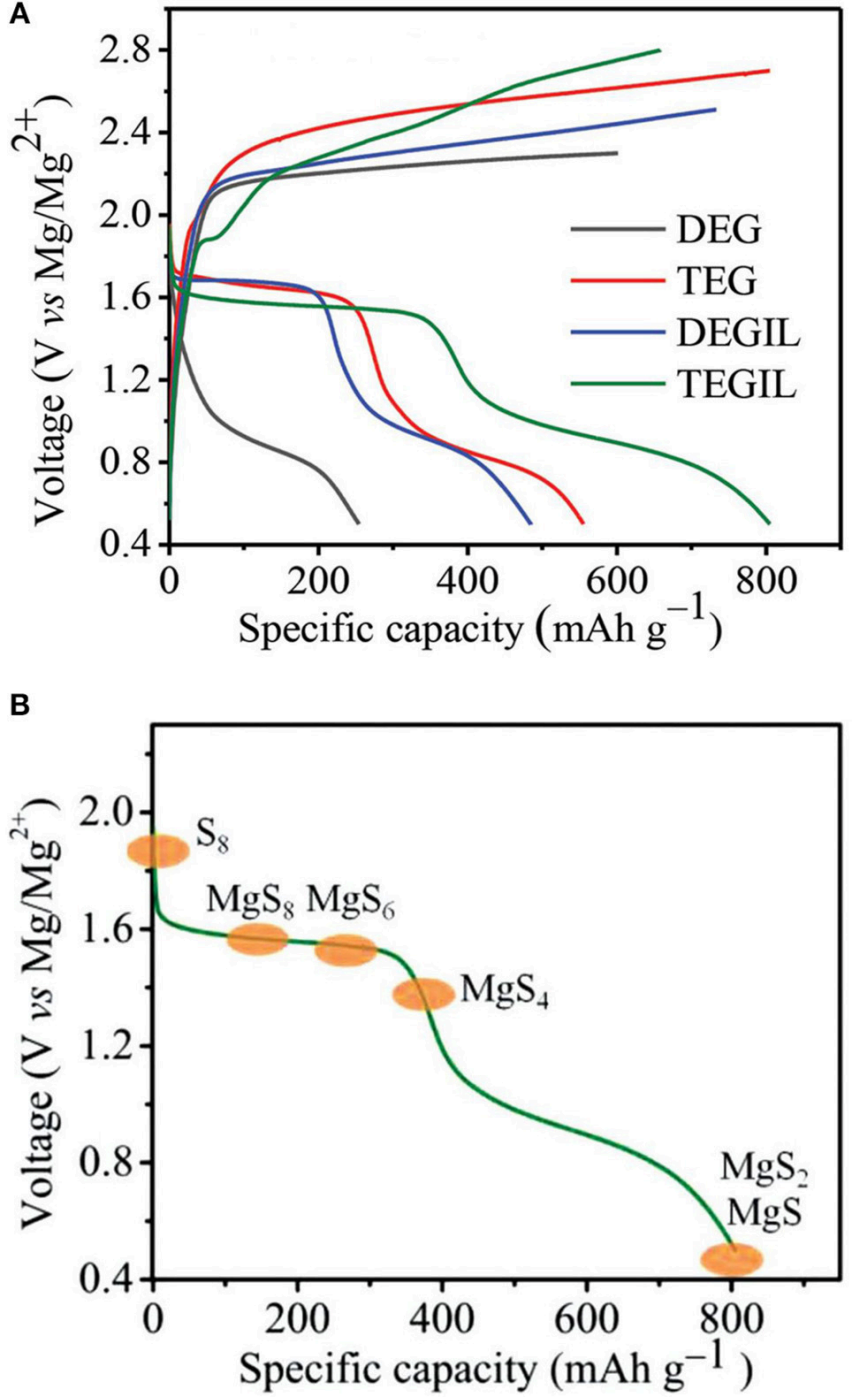
**(A)** Discharge/charge profiles of the Mg-S cells in Mg(HMDS)_2_-AlCl_3_ electrolytes. **(B)** Illustration of the redox mechanism in Mg-S batteries. Reprinted with permission from reference Zhao-Karger et al. (2015). Copyright (2015) WILEY-VCH Verlag GmbH and Co. KGaA, Weinheim.

The capability of reversible Mg deposition of the non-nucleophilic Hauser base hexamethyldisilazide magnesium chloride (HMDSMgCl) was first reported by Liebenow et al. (2000). With the modified HMDSMgCl-AlCl_3_ electrolyte in THF, Kim et al. presented the proof-of-concept of a Mg-S battery, where the cell could be discharge and charge for a few cycles with severe overcharging phenomena (Kim et al., 2011). We established a simple approach to formulate the non-nucleophilic bis-amide based electrolytes Mg(HMDS)_2_-AlCl_3_ in various ethereal solvents with superior electrochemical characteristics in terms of high oxidative stability and high coulombic efficiency (CE) for Mg plating/stripping (Zhao-Karger et al., 2013). With this type of electrolyte solutions in glymes, the cycling performance of the Mg-S batteries was greatly improved. The cell showed an open circuit voltage (OCV) of about 2 V and displayed two discharge plateaus, in which the flat upper discharge voltage plateau at 1.65 V is close to the emf, delivering an initial capacity of 800 mAh g^−1^ (Figure 1A) (Zhao-Karger et al., 2015). The cells retained a reversible capacity of about 260 mAh g^−1^ after 20 cycles. The reaction mechanism for the Mg-S cell was investigated through spectroscopic analysis, revealing the redox chemistry of sulfur involves two main steps: a fast solid–liquid two phase reduction forming high order polysulfide (MgS_8_, MgS_6_, MgS_4_), followed by liquid–solid reduction forming insoluble MgS_2_ and MgS (Figure 1B).

This study also indicates that the Mg-S system suffers from severe problems of rapid capacity degradation, poor charging efficiency and large charge over-potential, which possibly can be ascribed to the detrimental effect of the dissolved polysulfides on the properties of the electrolytes and/or the sluggish electrochemical reactivity of insoluble MgS_2_/MgS species (Zhao-Karger et al., 2015). Gao et al. introduced lithium bis(trifluoromethanesulfonyl)imide (LiTFSI) in the abovementioned Mg bisamide based electrolyte to enhance the reversibility of the Mg-S cell (Gao et al., 2015). The cell was demonstrated with two discharge plateaus at 1.75 and 1.0 V and a stable capacity of 1,000 mAh g^−1^ after 30 cycles. It was assumed that Li^+^ either undergoes an ion exchange reaction with MgS and MgS_2_, forming rechargeable Li_2_S and Li_2_S_2_, or reacts with MgS and MgS_2_ to form the long chain polysulfide MgLi-PS, thereby decreasing the kinetic barrier for re-oxidation of MgS_2_ and MgS (Gao et al., 2015).

**Figure 2:**
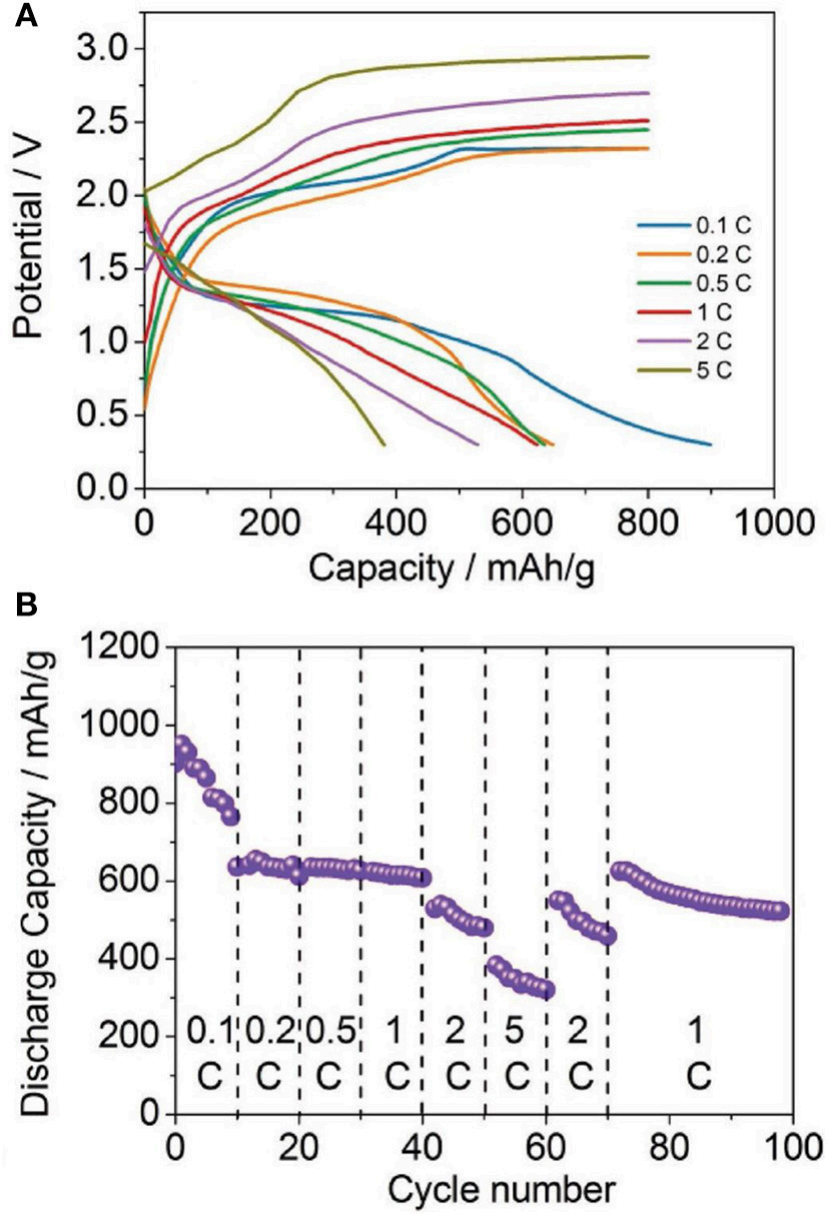
**(A)** Discharge/charge profiles, **(B)** cycling performances of the Mg-S cells at different current rates. Reprinted with permission from reference Zhou et al. (2018). Copyright (2018) WILEY-VCH Verlag GmbH and Co. KGaA, Weinheim.

As the sulfur chemistry in Mg based battery appears to be similar as in the Li-S system, the advanced strategies developed for Li-S batteries can be basically adopted to Mg-S system for performance enhancement. Yu et al. presented a cell design with a cathode comprising activated carbon nanofibers (CNFs) filled with sulfur powder and a CNF-coated separator (Yu and Manthiram, 2016). In their study, the Mg-S cells with the bisamide-based electrolyte delivered a high initial discharge capacity of ~1,200 mAh g^−1^ and lasted for 20 cycles without suffering a fast drop in capacity after the first cycle. A sulfur nano-composite was fabricated as cathode material by dispersing sulfur on the highly functionalized reduced graphene oxide (rGO) and improved reversible capacity upon cycling of the Mg-S batteries (Vinayan et al., 2016). Very recently, high-rate Mg-S batteries have been demonstrated using a MOF-based sulfur cathode incorporated with the bis-amide based electrolyte containing LiTFSI as additive, where a remarkable reversible capacity of about 400 mAh g^−1^ after more than 200 cycles at a rate of 1 C and a capacity of about 300 mAh g^−1^ at a high rate up to 5 C was achieved (Figure 2) (Zhou et al., 2018).

The bis-amide electrolytes have also been employed for magnesium batteries with selenium (Se) and selenium-sulfur (SeS_2_) cathode, respectively (Zhao-Karger et al., 2016). Owing to the intrinsic good electrical conductivity of Se, the cathode delivered a reversible capacity of 480 mAh cm^−3^ for 50 cycles at a high rate of 2C. In addition, SeS_2_ has also been tested as a high-capacity cathode material for Mg batteries, which offers the advantage to combine the merits of both selenium and sulfur (Zhao-Karger et al., 2016). Despite the feasibility and improvement for Mg-S and Mg-Se batteries has been demonstrated with the non-nucleophilic bis-amide electrolytes, it is also indicated that the Mg-S system still encounters critical problems such as the large voltage hysteresis, capacity fade upon cycling, and unsatisfied rate ability. The bis-amide electrolytes based on Mg(HMDS)_2_-AlCl_3_ as other Lewis base-acid combinations such as the known so called “APC” (all phenyl complex, PhMgCl-AlCl_3_) electrolytes are generally comprised of binuclear [Mg_2_(μ-Cl)_3_(THF)_6_]^+^ cation and aluminate anions in equilibria and their initial electrochemical properties could be severely deteriorated by the dissolved cathode or intermediates species (Zhao-Karger et al., 2013, 2014; Wan and Prendergast, 2014). In addition, the electrolytes containing chlorine (Cl) are corrosive to the metallic current collectors and other battery components and may also negatively influence the battery performance (Qiang et al., 2012; Wall et al., 2014), which restricts their practical applications. Therefore, a non-corrosive Mg electrolyte with proper electrochemical properties is required for the realization of practical Mg batteries.

#### With Mg-Ion Conductive Salt Based Electrolytes

**Figure 3:**
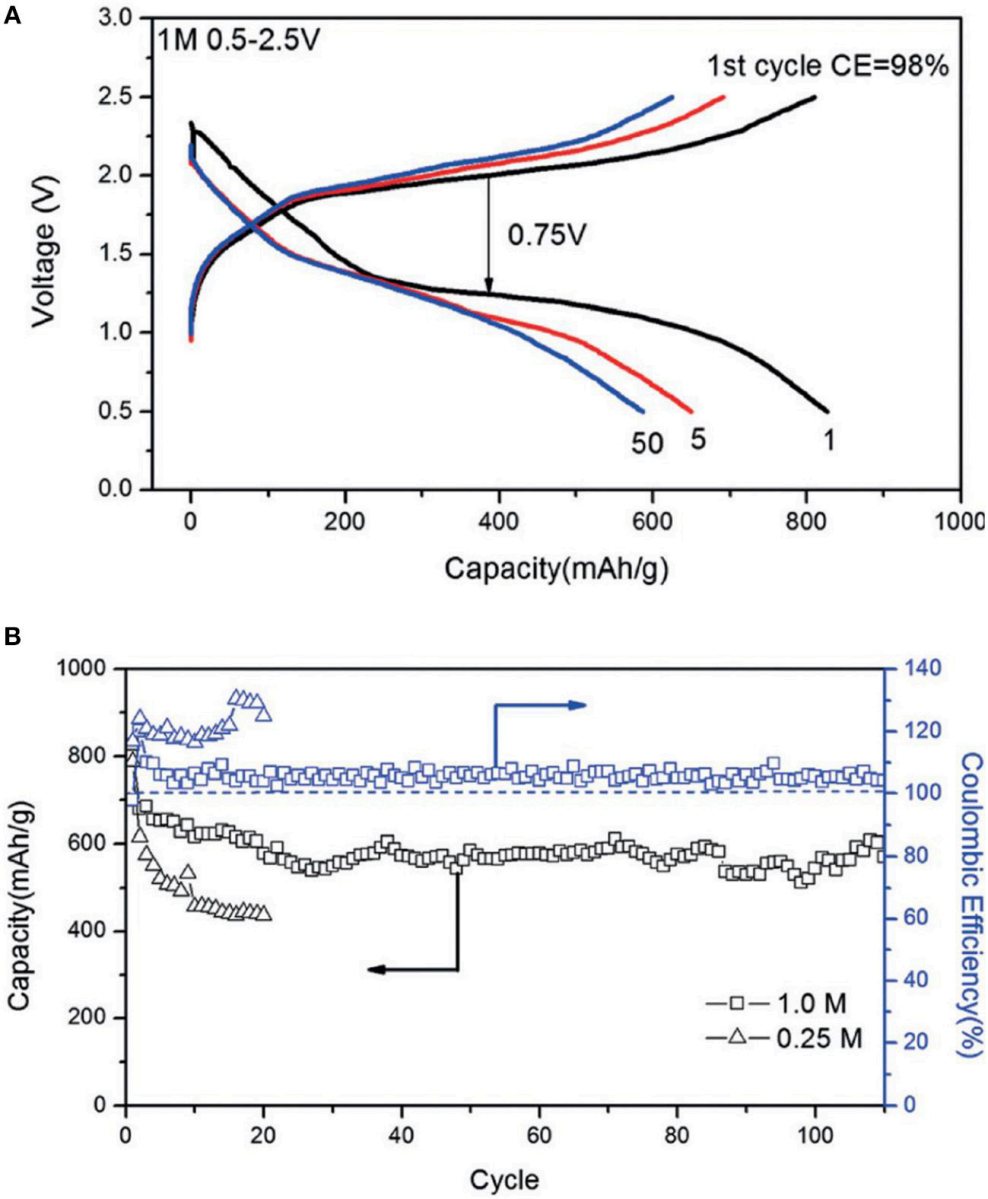
**(A)** Discharge/charge profiles, **(B)** cling performance of the Mg-S cells in Mg(TFSI)_2_-MgCl_2_/DME electrolytes at 0.1 C. Reprinted with permission from reference (Gao et al., 2017). Copyright (2017) WILEY-VCH Verlag GmbH & Co. KGaA, Weinheim.

Magnesium bis(trifluoromethylsulfonyl)imide [Mg(TFSI)_2_] in glymes was attempted for Mg-S batteries, where a poor cell performance was shown (Ha et al., 2014). Gao *et al* recently studied the thermodynamics of the Mg-S cell with the solution of Mg(TFSI)_2_ in dimethoxyethane (DME), in which the cells were operated at a low current rate (Gao et al., 2015). In a new approach, using the Mg(TFSI)_2_-2MgCl_2_ in DME, the cyclability of the Mg-S cell was significantly enhanced and a discharge capacity of about 600 mAh g^−1^ could be retained at 0.1 C after 100 cycles (Figure 3) (Gao et al., 2017). This enhanced battery performance could be ascribed to improved electrolytic properties of the Mg(TFSI)_2_ solution with additional MgCl_2_, in which the new formation of the electrochemical active MgCl^+^ species enables the effective reversible Mg deposition and the Cl^−^ ligand was supposed to play crucial role to refresh the Mg surface (Canepa et al., 2015; Shterenberg et al., 2015).

Owing to the inorganic composition and ready commercial availabilities, the combination of Mg(TFSI)_2_ and MgCl_2_ has been the preferred electrolyte formulation for Mg-S batteries in some studies. The mechanisms of the Mg-S redox reactions in Mg(TFSI)_2_-MgCl_2_ electrolyte in DME blended with 1,3-Dioxolane (DOL) has been studied by means of in operando X-ray diffraction (XRD) and K-edge x-ray absorption spectroscopy (XANES) (Robba et al., 2017). However, the in-depth studies revealed that a high reversibility of Mg deposition in this type of electrolytes could only be achieved through a chemical or an electrochemical conditioning process with additional Bu_2_Mg or application of voltage scans, respectively (Shterenberg et al., 2015). The macro-reversibility measurements indicated a steady increase in the over-potentials over prolonged Mg deposition/dissolution cycling, which is most likely due to the passivation on the Mg surface (Shterenberg et al., 2015). In this respect, other investigation on the alteration of the Mg surface in Mg(TFSI)_2_-2MgCl_2_ electrolyte identified the decomposition of TFSI^−^ anions and passivation of the anode at a practical current density of 1 mA cm^−2^ (Yoo et al., 2017). Thus, to evaluate the applicability of an electrolyte, the interfacial properties between the electrolyte and electrode should be carefully taken into consideration. In metal-sulfur batteries, it is particularly important to examine the surface alteration of the metal anode due to the known “shuttle” phenomena of polysulfides. Very recently, Salama et al. reported that the polysulfide species lead to detrimental passivation on Mg anode surface in the Mg-S system with Mg(TFSI)_2_-2MgCl_2_/DME electrolyte (Salama et al., 2018).

**Figure 4:**
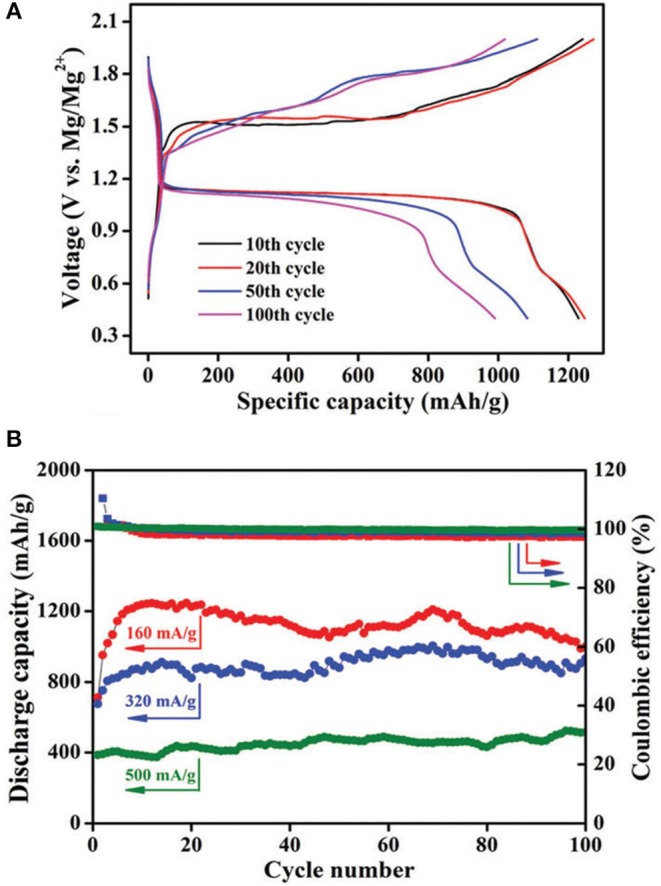
**(A) G**galvanostatic charge–discharge profiles of the Mg-S-CNT cell with the electrolyte [B(HFP)_3_]-Mg-MgCl_2_/DME at 0.1 C; **(B)** Discharge capacities and Coulombic efficiencies at different current rates. Reproduced from reference (Du et al., 2017) with permission from the Royal Society of Chemistry.

Zhang et al. prepared boron-centered anion-based magnesium (BCM) electrolyte and probed its suitability for Mg-S and Mg-Se batteries (Zhang et al., 2017). It is worth noting that the reversible Mg deposition of BCM electrolytes can only be generated after some potential scans as a “conditioning” step in the CV measurements, and increase in the concentration of Mg-ion in this type of electrolytes was observed. Nevertheless, a first discharge capacity of 1,081 mA h g^−1^ was measured using the electrolyte with an extremely low initial concentration of 0.05 M. In another approach, the same group modified the electrolyte formulation through the reaction of tris(hexafluoroisopropyl)borate [B(HFP)_3_] with Mg and MgCl_2_, where the S-CNT cathode could provide a relatively stable reversible capacity of about 400 mAh g^−1^ at a current rate of 500 mA g^−1^ for more than 100 cycles (Figure 4) (Du et al., 2017). With both of these B(HFP)_3_ based electrolytes, the Mg-S cells exhibited a flat discharge voltage plateau at about 1.1 V and a lower charging over-potential (<0.4 V) compared to other reports. It needs to mention that the use of Cu current collector was shown to be beneficial for the Mg-S cell performance although the electrolyte was measured to be less stable on Cu. The corrosion issue caused by Cl^−^ ions in these electrolytes would be a drawback for their practical use.

**Figure 5:**
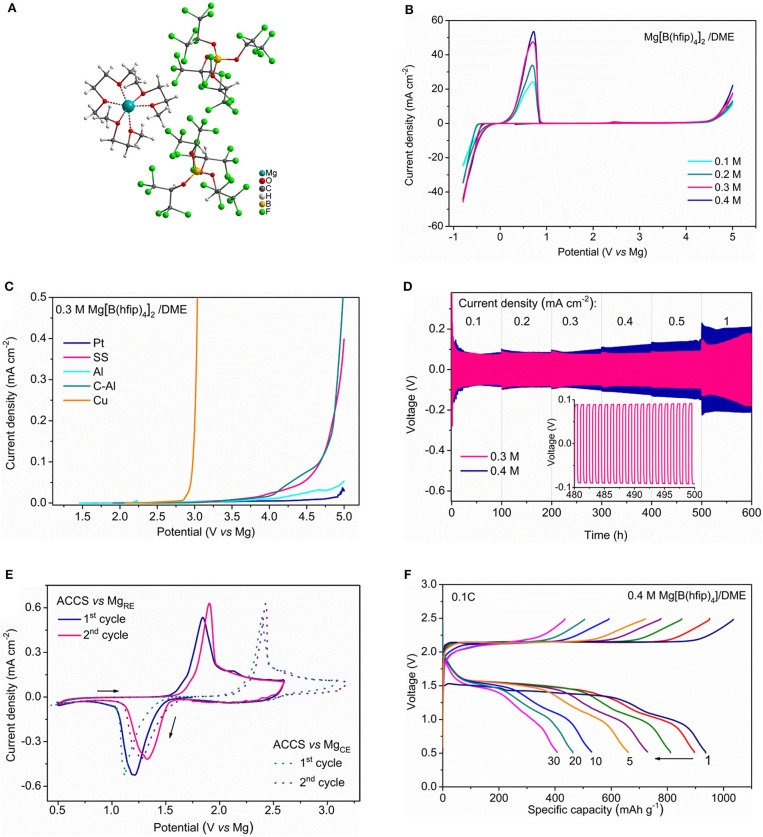
**(A)** X-ray crystal structure the Mg [B(hfip)_4_]_2_·3DME; Reprinted from reference (Zhao-Karger et al., 2017) with permission from the Royal Society of Chemistry. **(B)** Cyclic voltammograms on Pt electrode at a scan rate of 25 mV s^−1^. **(C)** Linear sweep voltammograms on various electrodes at a scan rate of 1 mV s^−1^. **(D)** Mg cycling performance of Mg[B(hfip)_4_]_2_/DME electrolytes in symmetric Mg|Mg cells at different current rates. **(E)** Cyclic voltammograms of the ACC-S electrode using Mg as both counter and reference electrodes. **(F)** Discharge/charge profiles of the Mg-ACCS cells in Mg[B(hfip)_4_]_2_/DME electrolyte at 0.1 C. Reprinted with permission from reference (Zhao-Karger et al., 2018) Copyright (2018) American Chemical Society.

The recent accomplishments in the highly efficient non-corrosive Mg ion-conductive salts provides new prospect for Mg batteries (McArthur et al., 2015, 2017; Tutusaus et al., 2015; Herb et al., 2016; Zhao-Karger et al., 2017; Hahn et al., 2018). The stable carba-closo-dodecaborate based electrolytes such as Mg(CB_11_H_12_)_2_ (MMC) is supposed to be non-nucleophilic but have not been tested for Mg-S battery so far. The ionic compounds of Mg fluorinated alkoxyborates and Mg alkoxyaluminates fulfill multiple requirements as electrolyte salts and can be universally incorporated with any type of cathode and anode materials (Zhao-Karger et al., 2017). For instance, the Cl-free Mg-ion conductive salt magnesium tetrakis(hexafluoroisopropyloxy) borate Mg[B(hfip)_4_]_2_ (hfip = OC(H)(CF_3_)_2_) has been employed as the highly efficient and non-corrosive electrolyte (Zhao-Karger et al., 2017). Recently, the bulk and interfacial properties of the Mg[B(hfip)_4_]_2_ electrolytes has been further investigated (Zhao-Karger et al., 2018). We demonstrated that the DME solutions of Mg[B(hfip)_4_]_2_ have the most promising electrolytic properties in current state-of-the-art Mg electrolytes in terms of high oxidative stability, high conductivity, low polarization and outstanding long-term cycling durability (Figure 5). Using a model sulfur cathode prepared with activated carbon-cloth (ACC) and the concentrated electrolyte solution of Mg[B(hfip)_4_]_2_ in DME, we demonstrate the highly reversible sulfur redox reactions in a Mg-based system with Mg[B(hfip)_4_]_2_ electrolyte. The discharge/charge profiles illustrate that discharge voltage remains at about 1.5 V for the extensive cycles at a current rate of 0.1 C. A discharge capacity of ~400 mAh g^−1^ was retained after over 30 cycles (Figure 5) (Zhao-Karger et al., 2018). Electrochemical and spectroscopic analyses verified the electrochemical reactions of the Mg-S system involving the transformation of a series of magnesium-polysulfide intermediates and also unveiled that the voltage hysteresis in Mg-S batteries origins from the half reaction at the anode (Zhao-Karger et al., 2018).

Compared to the Mg-S cells with bis-amide electrolyte, both of cyclability and Coulombic efficiency were improved by using the Mg[B(hfip)_4_]_2_ electrolyte and the overcharging problem was alleviated. However, the potential hysteresis between discharge and charge remains as an unsolved problem. In this respect, the properties of the electrolytes with additional Mg polysulfides was particularly examined (Zhao-Karger et al., 2018), in which the detrimental effect on Mg deposition/dissolution was confirmed and insoluble sulfide was also detected on the Mg surface. We believe that the degradation of both electrolyte and Mg anode caused by the polysulfides is the most critical issue which needs to be resolved before the Mg-S batteries can be regarded as a viable system.

### Mg-Halogen Batteries

The proof-of-concept Mg-Br_2_ battery has been demonstrated with a non-aqueous dual-electrolyte i.e., the catholyte comprised of Mg(TFSI)_2_ in glymes and the anolyte composed of Mg(TFSI)_2_ in an ionic liquid with additional MgBr_2_ (Yao et al., 2016). The cell was cycled for 20 times displaying a high discharge voltage of about 2.4 V. The battery performance could be possibly further improved through optimization of the cell setup and electrolyte formulation.

**Figure 6:**
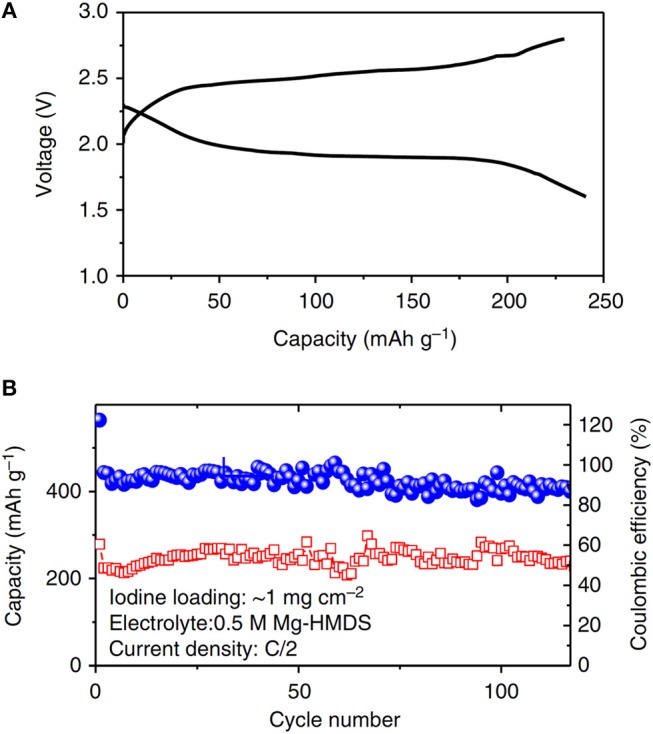
**(A)** Discharge/charge curve. **(B)** Cycling stability of the Mg-I_2_ batteries with bis-amide electrolytes. Reprinted with permission of Nature communications ^©^ 2017 Springer nature Ltd.

Iodine (I_2_) can be used as a conversion-type cathode material, where I_2_ can be reduced into a soluble intermediate tri-iodide followed by a rapid reversible two-electron reaction of the ${\text{I}}_{3}^{-}$/I ^−^ redox pair that can provide a theoretical capacity of 211 mA h g^−1^. With the bis-amide electrolyte, the couple of Mg-I_2_ has been demonstrated as a promising high power and high energy battery system (Tian et al., 2017). The battery could provide a working voltage of 2 V and a specific capacity of 180 mAh g^−1^ and 95% of the capacity could be retained at a rate of 0.5 C for 120 cycles (Figure 6). At a high-rate of 1 C, a discharge capacity of 140 mAh g^−1^ with an energy density of 400 Wh kg^−1^ was delivered.

Similar to the Mg-S chemistry, the liquid-solid two-phase reaction mechanism in Mg-I_2_ system is beneficial for the interfacial electron transfer to overcome the kinetic limitation of Mg^2+^ ion diffusion during charge and discharge the batteries. However, the soluble redox species in both systems may adversely lead to the shuttle phenomenon and a series of related problems such as self-discharge, low coulombic efficiency, and short cycle life.

## Organic Cathodes for Mg Batteries

**Scheme 1:**
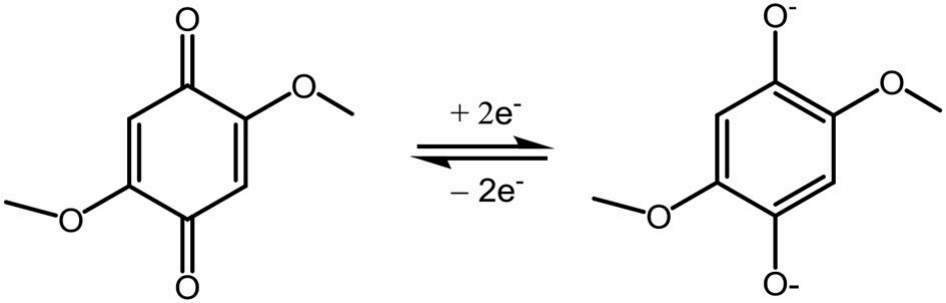
Electron redox mechanism of DMBQ.

Quinone-type compounds are the well-known organic cathode materials with carbonyl group as the redox center offering high capacity and fast reaction kinetics. This type materials can be categorized as conversion cathodes in that the double C = O bonds undergo a reversible enolization reaction forming C-O^−^ group through the stabilization of the conjugated molecular structures as shown in Scheme 1 with Dimethoxybenzoquinone (DMBQ) as an example.

Similar to sulfur, the electrophilic carbonyl moiety principally requires a non-nucleophilic Mg electrolyte to enable the reversible redox reactions. DMBQ was the first organic cathode probed in Mg battery systems. With the solution of Mg(ClO_4_)_2_ in γ-butyrolactone as electrolyte, the redox reaction was confirmed with a 3-electrode setup (Sano et al., 2012). Another approach was attempted by using the Mg(TFSI)_2_-sulfone solutions (Senoh et al., 2014). However, the cells exhibited a low discharge voltage and could run a few cycles, which could ascribe to the poor electrochemical properties of the Mg(TFSI)_2_ based electrolytes in terms of low reversibility of Mg deposition and large over-potential. Pan et al. examined the electrochemical properties of DMBQ in different electrolytes and verified that the electrolytes including Mg(BF_4_)_2_ in EC/PC, Mg(ClO_4_)_2_ in PC and Mg(TFSI)_2_ in diglyme with poor electrochemical properties results in inferior battery performance, e.g., low discharge voltage, large charge over-potential and low reversibility (Pan et al., 2016a). In contrast, the electrolyte of Mg(TFSI)_2_-2MgCl_2_ in DME enabled the Mg-DMBQ battery with a flat voltage plateau at 2.0 V and an initial discharge of 226 mAh g^−1^ (Pan et al., 2016a). However, rapid capacity fade was observed and a reversible capacity of 74 mAh g^−1^ was obtained after 30 cycles, which was assumed due to the inefficiency of the recharge and the high solubility of DMBQ in the electrolyte. Comparative study was conducted with organic polymer cathode poly(anthraquinonyl sulfide) (PAQS) and various non-nucleophilic electrolyte based on Mg(HMDS)_2_-AlCl_3_, MgCl_2_-AlCl_3_, and Mg(TFSI)_2_-MgCl_2_ (Bitenc et al., 2015). The Mg-PAQS cells exhibited a discharge voltage of 1.5 V and could deliver an initial discharge capacity close to the theoretical value of 225 mAh g^−1^ in Mg(TFSI)_2_-MgCl_2_ electrolyte with Mg powder anode. However, the capacity gradually dropped upon extended cycling and only <50 mAh g^−1^ was remained after 100 cycles (Bitenc et al., 2015). Poly(hydrobenzoquinonyl-benzoquinonyl sulfide) polymer (PHBQS) prepared from sulfur and hydroquinone has also been investigated for use as cathode for Mg batteries (Bitenc et al., 2016), where a discharge voltage of ~2.0 V and the maximum specific capacity of 158 mAh g^−1^ was abstained with the Mg(TFSI)_2_-MgCl_2_ electrolyte in a mixed solvent of tetraglyme and 1,3-dioxolane. The characteristic IR band of the C = O functional group allows the convenient analysis of the reversible electrochemical mechanism in the quinone-type cathodes (Vizintin et al., 2018).

**Figure 7:**
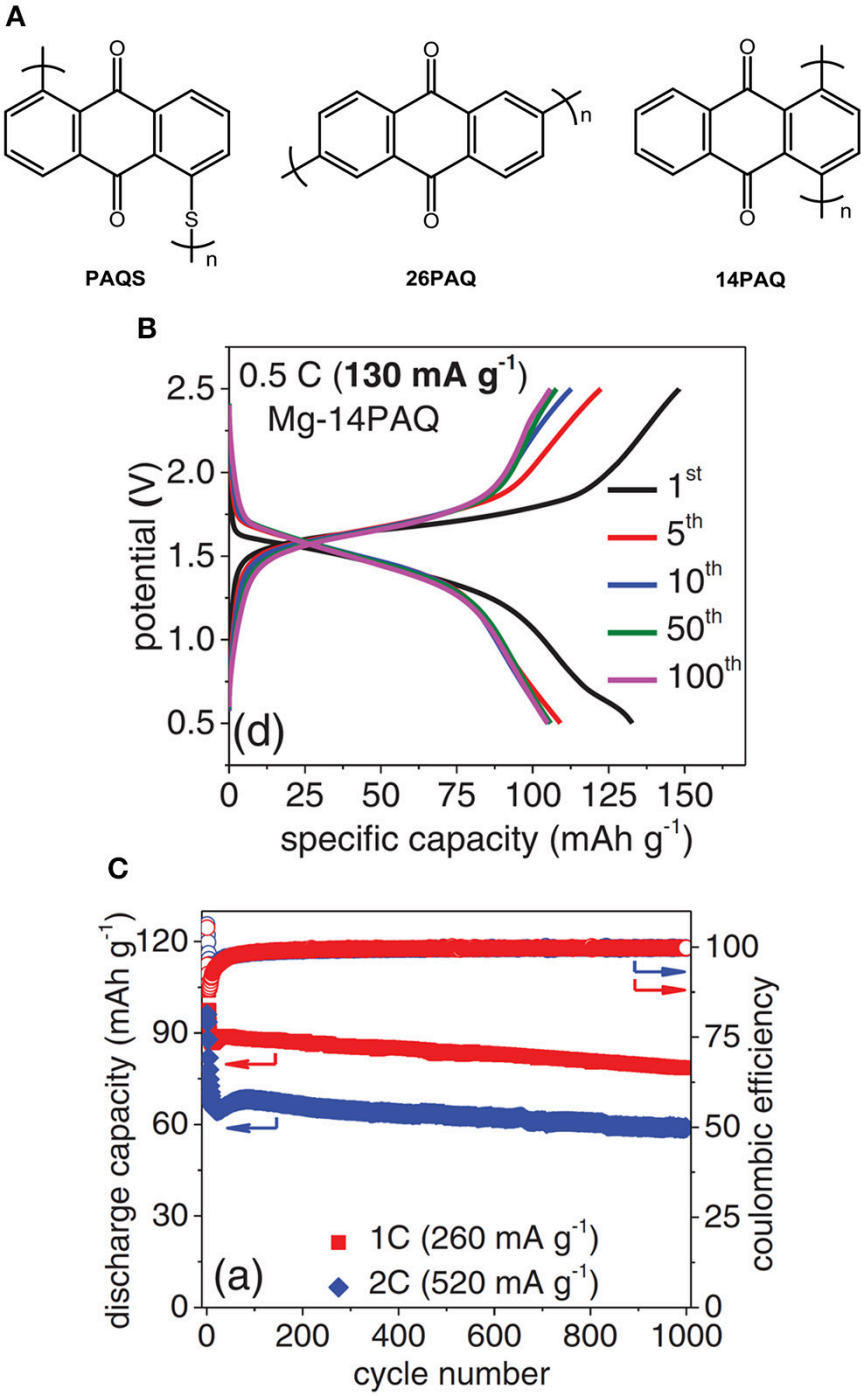
**(A)** Chemical structures **(B)** Discharge/charge curves, **(C)** cycling stability of Mg-14PAQ cells. Reprinted with permission from reference (Pan et al., 2016b). Copyright (2016) WILEY-VCH Verlag GmbH & Co. KGaA, Weinheim.

To effectively circumvent the dissolution of organic cathode materials, modern molecular design to tune the material properties has been demonstrated (Song et al., 2015). Poly2,6-anthraquinone (26PAQ) and Poly1,4-anthraquinone (14PAQ) was synthesized, respectively, as cathode materials with low solubility in organic electrolytes (Pan et al., 2016b). In particular, 14PAQ cathode in Mg(HMDS)_2_-MgCl_2_ electrolyte exhibited a superior performance compared to PAQS and 26PAQ in terms of low potential hysteresis, high reversible capacity and stable cycling stability. The Mg-14PAQ cells displayed a discharge voltage of 1.6 V and can be operated up to 1,000 cycles with a Coulombic efficiency >99% at a rate of 1 C (Figure 7) (Pan et al., 2016b).

A naphthalene-hydrazine diimide polymer (NP) has been tested as a cathode material for Mg organic batteries in two different electrolytes i.e., Mg(TFSI)_2_-MgCl_2_ in tetraethylene glycol dimethyl ether (TEGDME) and a mixture of TEGDME and 1.3-dioxlane (DOL) (1:1, v/v) (Bančič et al., 2018). In both electrolytes, an average discharge voltage of about 1.7 V was exhibited and a discharge capacity of 150 mAh g^−1^ was achieved, which is around 42% of theoretical capacity (364 mAh g^−1^) for NP. The cells using the electrolyte in TEGDME/DOL showed superior electrochemical behavior with slow capacity fading and cycling good Coulombic efficiency (99%).

**Figure 8:**
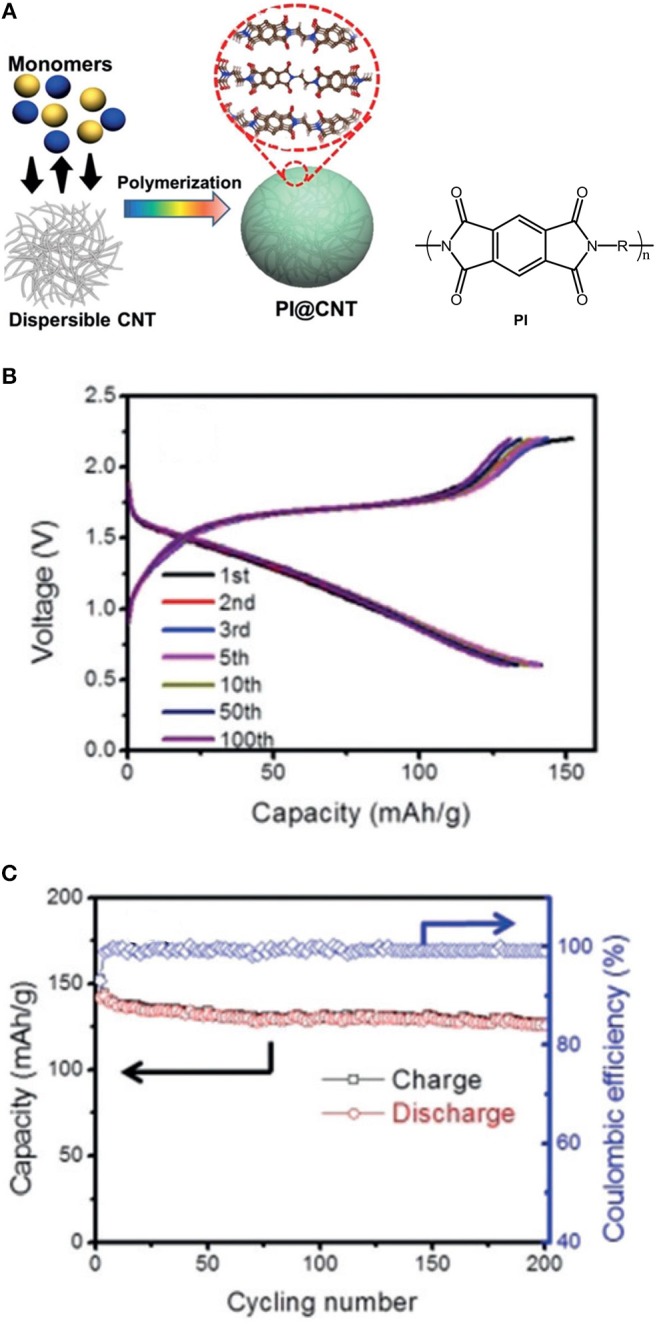
**(A)** Preparation of PI@CNT composite. **(B)** Charge/discharge profiles, **(C)** cycling performance of the Mg-PI batteries at 1 C. Reprinted with permission from reference (Fan et al., 2018). Copyright (2016) WILEY-VCH Verlag GmbH & Co. KGaA, Weinheim.

Recently, a new polyimide (PI) based cathode material for both Mg and Al battery systems has been demonstrated (Fan et al., 2018). The preparation process of the PI@CNT composite is shown in Figure Xa. The PI@CNT cathodes provide a highly reversible capacity of about 130 mAh g^−1^ (based on the weight of the composite) at 1C for over 200 cycles in the Mg batteries using THF solution of phenylmagnesiumchloride-AlCl_3_ as electrolyte (Figure 8). It is worthy to mention that no capacity decay was observed even after 10,000 cycles for the Li based batteries with a 4 M LiFSI/DME electrolyte and the cycle life of the Mg-PI cells could be expected to be further enhanced by choosing a more efficient electrolyte.

## Conclusion and Outlook

To summarize, conversion-type cathodes with intrinsically favorable redox kinetics can be considered as promising candidates for high-energy Mg batteries. The development of Mg-S batteries is being facilitated through recent advanced electrolytes. However, the current research results of Mg-S batteries were obtained under different cell conditions, especially with different types of electrolytes and the cathode fabrications, which make it difficult to draw a proper conclusion to decide a better electrolyte or cathode so far. In general, for the assessment of the applicability of an electrolyte for rechargeable batteries, it is essential to examine not only the electrochemical properties but also the long-term cycling and the interfacial behaviors between the electrolyte and electrodes. In this respect, the non-corrosive conductive salt Mg[B(hfip)_4_]_2_ has been shown to be promising as the practical electrolyte for Mg batteries. Nevertheless, comprehensive knowledge in the reactions at the electrode-electrolyte interface and the chemistries taking place in Mg-S battery systems are of paramount importance for developing strategies to overcome the remaining limiting factors. One of the key issues for the current research stage of Mg-S battery appears to be Mg anode passivation and electrolyte degradation mainly stemming from the dissolution of polysulfide similar to other metal-sulfur systems. In the light of the advancement in the development of Li-S and Na-S batteries, some approaches can be considered for Mg systems, including employing electrolyte additives for protection or activation of Mg anode, effectively encapsulating sulfur within the cathode, functionalizing the separator to prevent the migration of polysulfides to the anode side.

In addition, organic materials are of great potential to be used as high capacity cathode for Mg batteries. The main drawbacks of organic compounds such as high solubility and low electric conductivity could be circumvented by molecular design and synthetic approaches. One of the particular advantages of organic electrodes for Mg batteries is that they are structurally diverse and their properties can be tuned by modifying the chemical structure, which provide an excellent opportunity to overcome some fundamentally limitations of Mg-ion transport achieving high-performance Mg batteries.

## Author Contributions

ZZ-K conceived the concept of this review and wrote the manuscript. All authors discussed and commented on the manuscript.

### Conflict of Interest Statement

The authors declare that the research was conducted in the absence of any commercial or financial relationships that could be construed as a potential conflict of interest.
